# Synonymous Co-Variation across the E1/E2 Gene Junction of Hepatitis C Virus Defines Virion Fitness

**DOI:** 10.1371/journal.pone.0167089

**Published:** 2016-11-23

**Authors:** Brendan A. Palmer, Liam J. Fanning

**Affiliations:** Molecular Virology Diagnostic & Research Laboratory, Department of Medicine, University College Cork, Cork, Ireland; Tel Aviv University, ISRAEL

## Abstract

Hepatitis C virus is a positive-sense single-stranded RNA virus. The gene junction partitioning the viral glycoproteins E1 and E2 displays concurrent sequence evolution with the 3′-end of E1 highly conserved and the 5′-end of E2 highly heterogeneous. This gene junction is also believed to contain structured RNA elements, with a growing body of evidence suggesting that such structures can act as an additional level of viral replication and transcriptional control. We have previously used ultradeep pyrosequencing to analyze an amplicon library spanning the E1/E2 gene junction from a treatment naïve patient where samples were collected over 10 years of chronic HCV infection. During this timeframe maintenance of an in-frame insertion, recombination and humoral immune targeting of discrete virus sub-populations was reported. In the current study, we present evidence of epistatic evolution across the E1/E2 gene junction and observe the development of co-varying networks of codons set against a background of a complex virome with periodic shifts in population dominance. Overtime, the number of codons actively mutating decreases for all virus groupings. We identify strong synonymous co-variation between codon sites in a group of sequences harbouring a 3 bp in-frame insertion and propose that synonymous mutation acts to stabilize the RNA structural backbone.

## Introduction

Hepatitis C virus (HCV) is genetically diverse. Within the last decade, one new HCV genotype (genotype 7) and 49 new subtypes have been defined [1, 2]. This genetic diversity is founded upon the virally encoded, highly error prone RNA-dependent RNA polymerase [3]. Each HCV genome replication cycle is projected to contain one random mutation event [3, 4]. The accommodation of defined hypervariable regions (HVR) within the HCV genome ensures significant sequence diversity can also be observed at the within-host level. Even at the single nucleotide level, individual sites demonstrating high mutation and low mutation rates have been documented [5].

The potential for exploration of the sequence space is vast, yet collapses in genetic diversity longitudinally have been documented. This may be as a result of the masking of minor variants by singly dominant sequences, giving the semblance of a clonal phenotype [6]. In one example, analysis of a chronic HCV genotype 4a patient sample yielded just five unique HVR1 amino acid motifs from more than 15,000 individual sequence reads [7]. All five were interconnected by a genetic distance of one amino acid. It is therefore pertinent to examine not only the breadth of the sequence space explored by a virus, but also the apparent limitations behind a lack of exploration [8].

There is increasing evidence that synonymous mutations, which do not alter the amino acid profile, can also have a significant impact on the fitness of viral populations [9, 10]. In a quasispecies of closely related sequences, synonymous codon selection places individual variants in different regions of the sequence space and subsequent changes at these sites can lead to distinct evolutionary trajectories. There is increased recognition that epistasis, the effect(s) of a mutation on the presence or absence of other mutations in the genome, is an important facet of viral fitness. HIV-1 treatment resistance mutations have been observed to occur in tandem with subsequent compensatory mutations to correct for consequential loss of fitness [11]. A number of studies have indicated that significant fitness effects can result solely as a consequence of synonymous mutation [10, 12–14].

In this study, we take advantage of a previously reported data set that is ideally suited to explore epistatic change over an extended period [7]. Firstly, the data spans samples collected over a 10 year period from a HCV chronically infected, treatment naïve patient. Secondly, the sample space was initially comprised of two lineages (L1 and L2) that fluctuated in their dominance of the virome. L1 was of sufficient complexity that sub-lineages L1a, L1b and L1c could be defined. L1 sequences dominated the virome interchangeably for the first 8.6 years of the sampling period (samples 1–8). Each sub-lineage gained temporal dominance within the host and L1a, L1b and L1c were subjected to IgG targeting by the humoral immune response [7]. In addition, towards the latter sampling points, L1 sequence frequency dropped below the levels of detection allowing L2, which was of low genetic diversity and lacked IgG targeting, to rise and dominate the virome *in toto*. Thirdly, L1b sequences contained an in-frame 3 bp insertion within the HVR1. Fourthly, the amplicon spans the E1/E2 gene junction and represents protein elements that are subject to distinct evolutionary pressures. The C-terminal end of E1 forms part of the trans-membrane domain of the protein that engages with capsid protein in the mature virion, whereas the N-terminal end of E2 encodes the antigenic HVR1 [15]. Finally, the sequence set was obtained using ultradeep pyrosequencing. Application of a temporally matched clonal dataset to complement the error correction methodology allowed for considerable sequence depth to be reached [7, 16].

We show disparate patterns of co-variation amongst mutating codon pairs for L1a and L1b sequence subsets. We report that nonsynonymous epistasis dominated the L1a sequence subset and was linked to HVR1 variant change while synonymous co-variation enhanced fitness. We find that synonymous co-variation defined the L1b sequence subset and hypothesize that accommodation of the insertion curtailed mutational flexibility.

## Results

### Sample set overview and background

The sample set used for this study comprised of ten serum samples from a single, treatment naïve patient, chronically infected with HCV genotype 4a, that were collected over 9.6 years [7]. The mean time between samples was 1.07 years (sd ± 0.43 years). Phylogenetically, the sequence set partitioned into two main lineages named L1 and L2. L1 could be divided further into three sub-lineages, namely L1a, L1b and L1c, based on bootstrap values >85 for each of the main branches [7]. The emergence, dominance and decline of (sub-)lineages are outlined in S1 Table for reference. L1c sequences were recovered from just 3/10 samples and due to the limited sequence availability were omitted from this analysis. Overall, for L1a and L1b, codon switches were observed to occur at a frequency of 0.071 and 0.079 per site per sequence, respectively. The codon mutation rate for L2 sequences by contrast was 0.028 per site per sequence.

### Codon fixation increased over time for all sequence groupings irrespective of sample frequency

**Fig 1:**
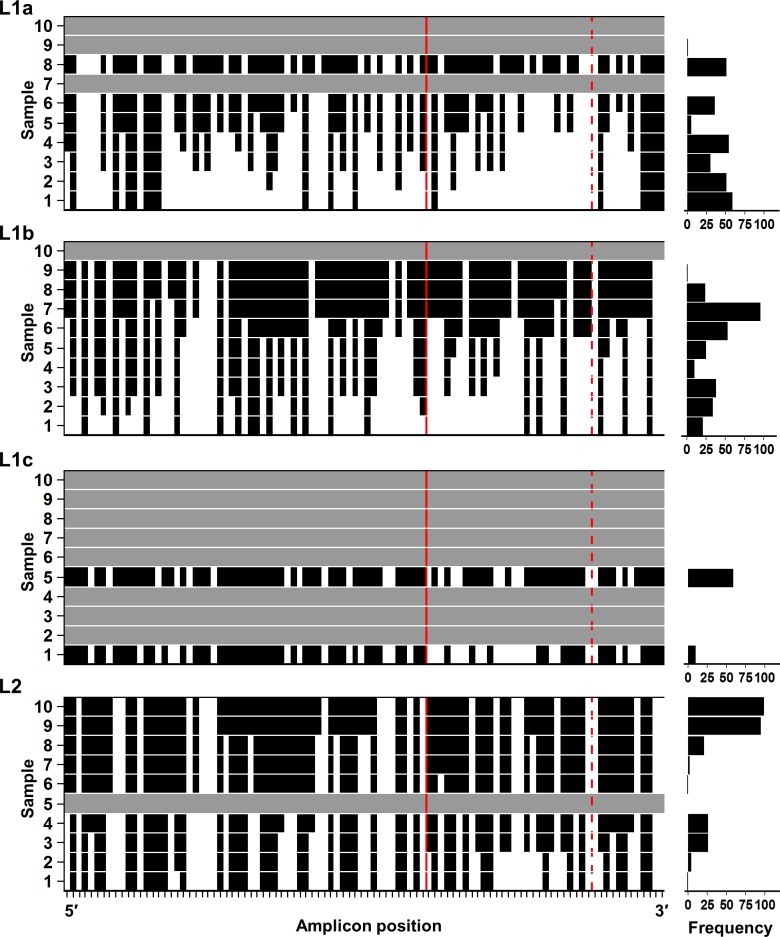
Within-lineage codon fixation during the study timeframe of the L1a, L1b, L1c and L2 sequence subsets. Left panel: For the purposes of this analysis, a site is designated as fixed (black) when a single codon accounts for all the sequences in that sample and all subsequent samples thereafter. For all sequence subsets overtime, the proportion of codon sites actively mutating decreased across the length of the amplicon including the HVR1. Notably, just 3/27 L1b HVR1 codon sites displayed ongoing codon switching events post sample 7. L2 contained the highest proportion of sites that were invariant throughout the sampling timeframe. In spite of the sample space expansion of L2 between samples 8–10, the number of fixed codon sites increased overall. The E1/E2 gene junction and the last codon of the HVR1 are identified by a solid red line and a dashed red line, respectively. Samples with absent or insufficient sequence data (less than two unique sequences) are shaded as grey horizontal bars. Tick marks along the X-axis identify each codon position of the amplicon sequence. Right panel: The sample specific frequency of each (sub-)lineage.

Sequences, grouped by (sub-)lineage, were examined at each codon position to identify the mutational flexibility across the length of the amplicon during the study timeframe. L2 contained 42/98 invariant codon sites overall which reflected the low mutation rate of this sequence set. This compares to 16/98 and 19/98 invariant sites within the L1a and L1b sequence subsets, respectively. Unexpectedly, following L2 expansion into the sample space, and despite an increase in the number of unique sequences isolated, the number of actively mutating codon positions decreased over time (Fig 1 and S1 Table).

**Fig 2:**
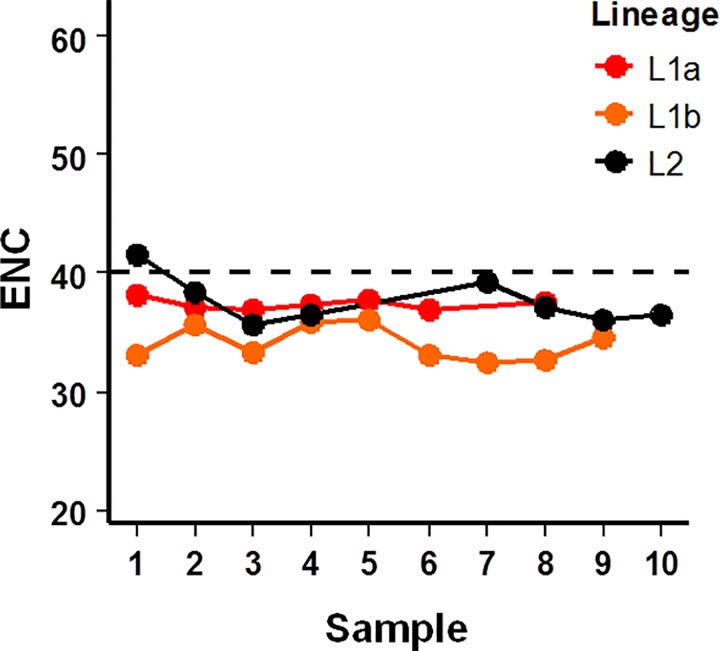
Effective number of codons utilized by each HCV (sub-)lineage overtime. Values less than the threshold of 40 (dashed line) are considered as biased utilization of the available redundancy within the genetic code.

It was apparent that the accommodation of the in-frame insertion into the HVR1 of L1b sequences negatively impacted on the mutational flexibility of the HVR1. L1b sequences accounted for 96.5% of all sample 7 isolates, yet at that time only four HVR1 codons were actively mutating (and only three thereafter) (Fig 1). This was further evidenced when codon usage frequencies were assessed. Evidence of codon usage bias was present in the data for each of L1a, L1b and L2 (Fig 2). L1b sequence ENC values were consistently lower over the 10 sampling points (minimum ENC 32.4).

### Differential patterns of epistasis define (sub-)lineage glycoprotein evolution

**Fig 3:**
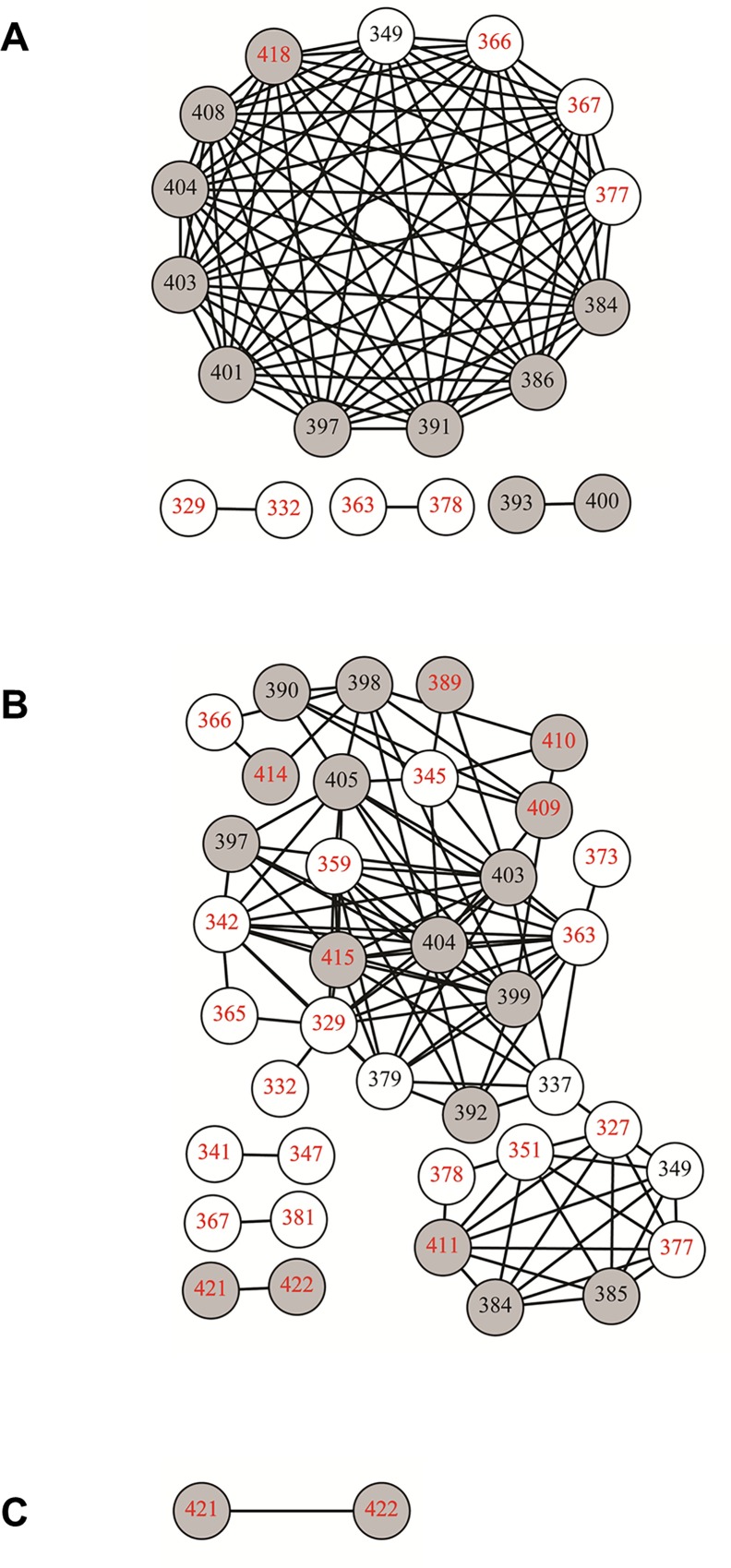
Pronounced epistasis was evident for both L1a and L1b sequence sets covering 10 years of continuous adaptive evolution of the E1/E2 gene junction. Nodes (representing codon positions) within the graph are connected by an edge if the probability of a change detected simultaneously at both sites was statistically significant (p-value < 0.01). (A) L1a epistasis was highly ordered with the majority of significantly linked sites participating in a single large connected component. (B) Epistasis within the L1b sequence set was observed among a greater number of codon sites overall. The majority of sites identified for L1b exclusively underwent synonymous mutation. (C) Two sites were observed in L2 sequences that were below the significance threshold. White nodes define codons within the E1 coding sequence, while grey nodes identify E2 codons. Sites containing nonsynonymous mutations are identified by black numbers while sites exclusively undergoing synonymous mutation are given by red numbers. Nodes are numbered in accordance with the amino acid positions of the H77 reference genome (Genbank accession: AF009606).

Co-variation between codon sites identified epistatic evolution within E1, within E2 and across the E1/E2 gene junction (Fig 3). It was observed that co-variation was deterministic with the same codon-codon switching events being replicated between unique sequences in the majority of instances (S2 and S3 Tables).

**Fig 4:**
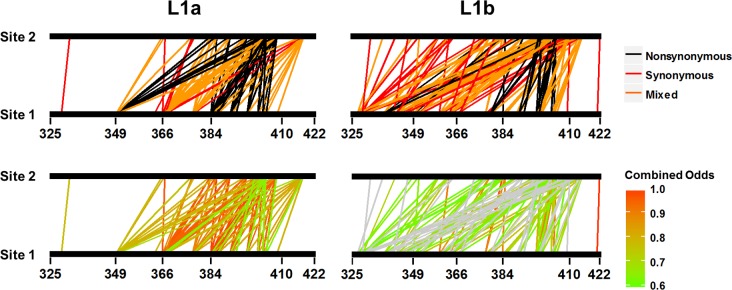
Bipartite mapping of co-evolving sites to the amplicon. Top panel: Co-evolving pairs are identified as nonsynonymous-nonsynonymous, synonymous-synonymous or a combination of one site mutating nonsynonymously and one site mutating synonymously. Bottom panel: The combined odds of a mutation co-varying with a mutation at a second site are given by the colored scale bar. Co-varying pairs represented by grey bars have combined odds <0.6 which indicates that, for a given sequence set, one of the two sites has a greater observed mutational flexibility than that observed at the second site. Raw data counts, individual odds and combined odds are provided in S4 and S5 Tables for reference.

Of the 19 co-varying sites that were identified as significant for L1a sequences, 13 formed a highly structured interconnected network. Indeed, all 13 sites had edges connecting to the remaining 12 sites (Fig 3A). This included nonsynonymous-nonsynonymous mutations that crossed the E1/E2 gene junction (codon 349, Figs 3 and 4). Within this network, four sites were synonymous (codon positions 366,367, 377 and 418) with the remaining nine nonsynonymous. Those sites with the greatest significance were nonsynonymous-nonsynonymous interactions within the HVR1 (S4 Table), which was in line with model assumptions.

A more complex and irregular pattern of co-variation was present amongst sites within the L1b sub-lineage (Fig 3B). L1b sites were distributed across the length of the amplicon when compared with the L1a sequence set (Figs 3 and 4). Thirty three sites exhibited significantly linked co-variation to at least one other site. Unlike L1a, the most significant values were not between nonsynonymous-nonsynonymous co-variation within the HVR1, but rather included synonymous change at one or both sites within E1 (Fig 4 and S5 Table). Phylogenetic analysis of just the E1 portion of the amplicon maintained the overall separation of L1a and L1b branches (data not shown). The sequence length was too short (177 bp) for strong phylogenetic inference to be made but this observation indicates that the presence of the insertion applied sequence specific mutational patterns to 3′-end of E1. Furthermore, the majority of L1b sites (19/33) were synonymously mutating throughout.

A single synonymous-synonymous epistatic event was present for L2 sequences at the 3′-end of the amplicon (Fig 3C, codon positions 421 and 422). No significant co-variation was detected in the reference data set using this methodology.

The strength of epistatic interactions contrasted between L1a and L1b sequences. The odds of a codon switching event at a co-evolving site participating in a paired mutational event was high for L1a (Fig 4 and S4 Table). In all instances the combined odds were >0.6. In contrast, a large number of mutations for L1b sequences at significant co-varying sites had an odds ratio <0.6. This observation suggests forced synonymous change to accommodate constraints introduced as a consequence of HVR1 motif drift.

**Fig 5:**
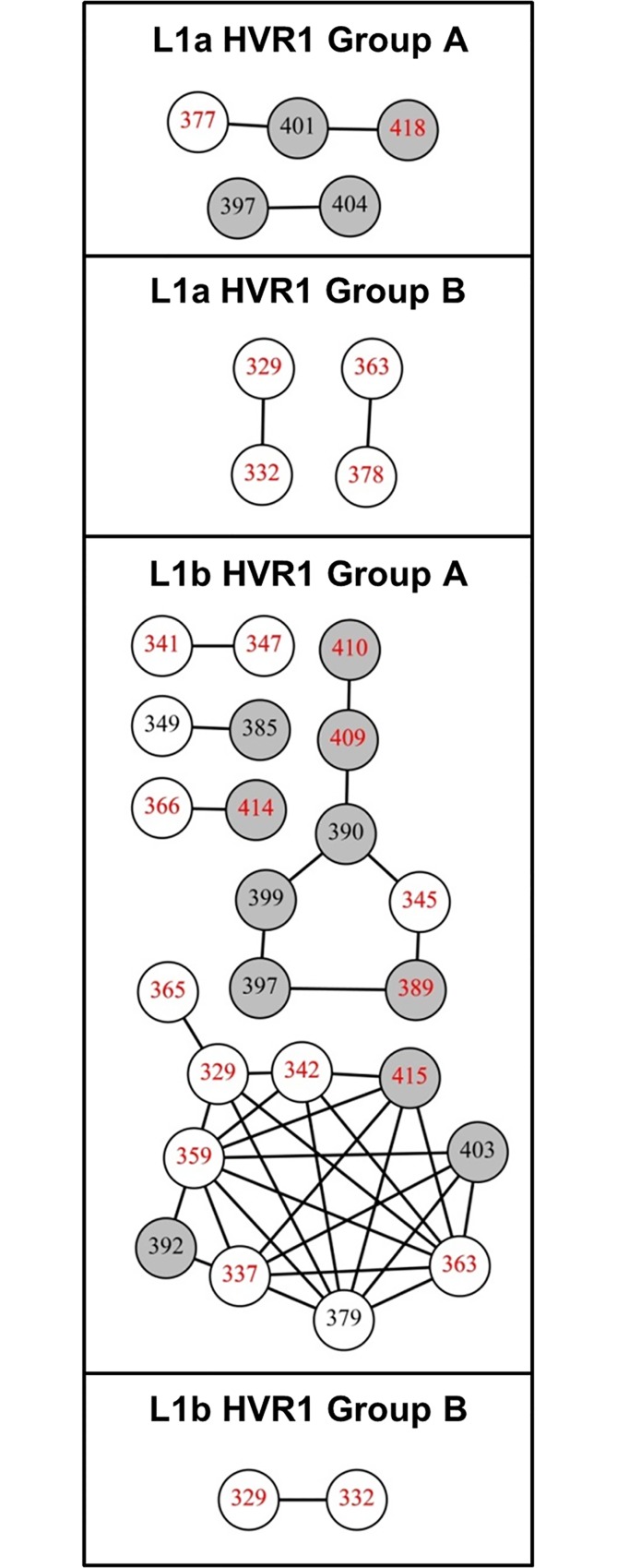
Sub-division of L1a and L1b sequences by HVR1 motif defined sequence stability. Significant co-variation between sites was determined separately for L1a sequences and L1b sequences split by HVR1 motif. White nodes define codons within the E1 coding sequence, while grey nodes identify E2 codons. Sites containing nonsynonymous mutations are identified by black numbers while sites exclusively undergoing synonymous mutation are given by red numbers. Nodes are numbered in accordance with the amino acid positions of the H77 reference genome (Genbank accession: AF009606).

The highly ordered network of epistatic evolution reported for L1a was underscored by the constituent HVR1 amino acid motifs which fell into two discrete groupings that demonstrated interchangeable dominance sample (S1 Fig). This is in line with the isolation of the same amino acid sequence across multiple samples [17]. L1a HVR1 groups A and B comprised 11 and 8 unique HVR1 amino acid motifs respectively. Within-group evidence of epistasis was limited indicating overall sequence stability (Fig 5).

L1b HVR1 amino acid motifs also partitioned into two groups containing 30 unique motifs (group A) and 9 unique motifs (group B). The closest detectable ancestor to the insertion event, L1a clone GQ985348 isolated from sample 1 [17], branches with L1b group A sequences. Erratic networks of epistatic evolution were detected for this group, which also exhibited the greatest amount of ongoing codon mutation across the length of the amplicon (Fig 4 and S1 Fig). The high proportion of synonymous mutations suggests constraints placed upon the sequence in order to accommodate the insertion initially. Virions harboring the L1b group A motif set never occupied more than 25% of the sample space (S1 Fig). A single codon within the E2 signal peptide (residues 371–383) was identified in nonsynonymous co-varying pairs (Figs 3B and 4, codon 379). However, nonsynonymous codon change accounted for less than 3% of all codon switching events that were recorded at this position and sequences containing the change accounted for just 0.2% of the sample space overall.

The elimination of L1b group A sequences post-sample 5 facilitated the establishment of a fitter, more genetically stable population (group B) that attained near absolute occupation of the sample space towards the latter end of the sample timeframe (S1 Fig). A single significant synonymous-synonymous co-evolving pair was observed for this sequence subset (Fig 5).

### Humoral immune evasion pathways were defined by sequence stability

**Fig 6:**
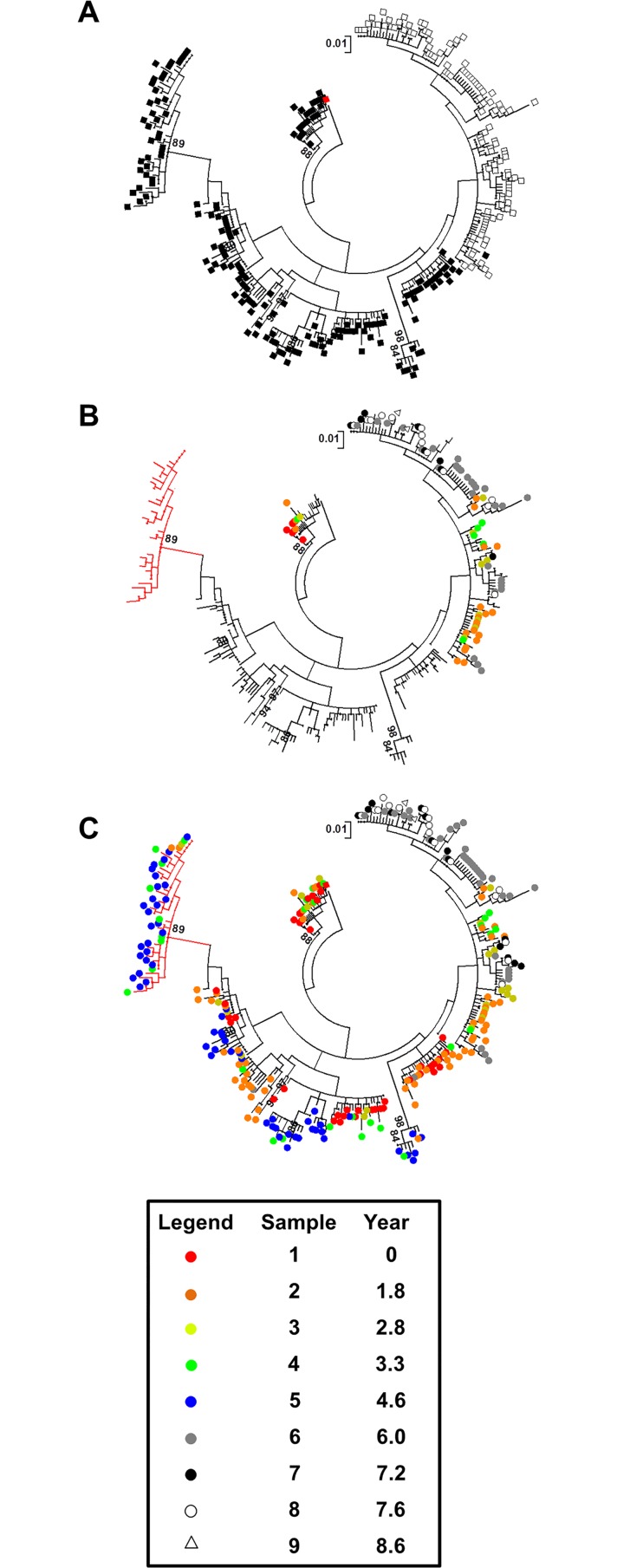
Mapping of the L1b sequence set in the context of HVR1 motif groups. All trees have been rooted using the nearest detectable ancestor to the insertion event, GQ985348 [red square, 17]. The sample specific isolation of each sequence is defined by the color legend. (A) The L1b sequence subset was split into two groups, A (black squares) and B (open squares), which were defined by the constituent HVR1 amino acid motifs. (B) Colored nodes identify those sequences coding for HVR1 motifs that have previously been associated with IgG-bound virions [7]. A single IgG-associated motif from group A was isolated from sample 1 (red circle). An additional IgG-associated motif for group B was isolated from sample 6 (grey circle), four years after the motif was first detected in whole patient serum. A phylogenetically diverse branch of L1b group A sequences not subject to detectable IgG binding is indicated by red branches. (C) Group A sequences not subject to IgG-targeting exhibited the greatest sequence diversity. Nevertheless, this subpopulation collapsed post-sample 5. The genetic distance is shown as a bracketed scale bar. Bootstrap values >80 of 1000 resamplings are shown. Genetic distance is given by the scale bar.

The separation of L1b sequences by HVR1 motif provided novel insights into the data. L1b group A sequences were phylogentically more diverse whereas group B occupied a narrower sequence space (Fig 6A). Two unique L1b HVR1 amino acid motifs were predicted from the isolation of IgG-bound virions [7]. One of each aligned with L1b group A and group B motifs. Significantly, the L1b group A variant was isolated from sample 1 and represented a small proportion of the overall number of L1b group A sequences (Fig 6B). L1b group A sequences represented 100% of all detectable L1b sequences by sample 5 (Fig 6C and S1 Fig). No evidence of immune targeting was observed for distal branches of L1b group A sequences during this period (Fig 6B).

In spite of this, and compounded by apparent fitness constraints, this subset of the virome collapsed post-sample 5. Interestingly, the L1b group B motif, identified from IgG-bound virions, was not detectable following fractionation of IgG-bound virions until sample 6. It was nevertheless present in unfractionated preparations as early as sample 2. This L1b group B variant was presumably masked from immune targeting by dominant co-circulating variants (Fig 6B and S1 Fig). As L1b group B variants rose to dominate the virome, escape mutations did not develop post-sample 6. All L1b group B variants contained the same HVR1 motif and this motif was associated with IgG-binding (Fig 6B). L1b was at the limit of detection by sample 9 and undetectable by sample 10.

### Predicted RNA structures suggest enhanced L1a sequence stability

**Fig 7:**
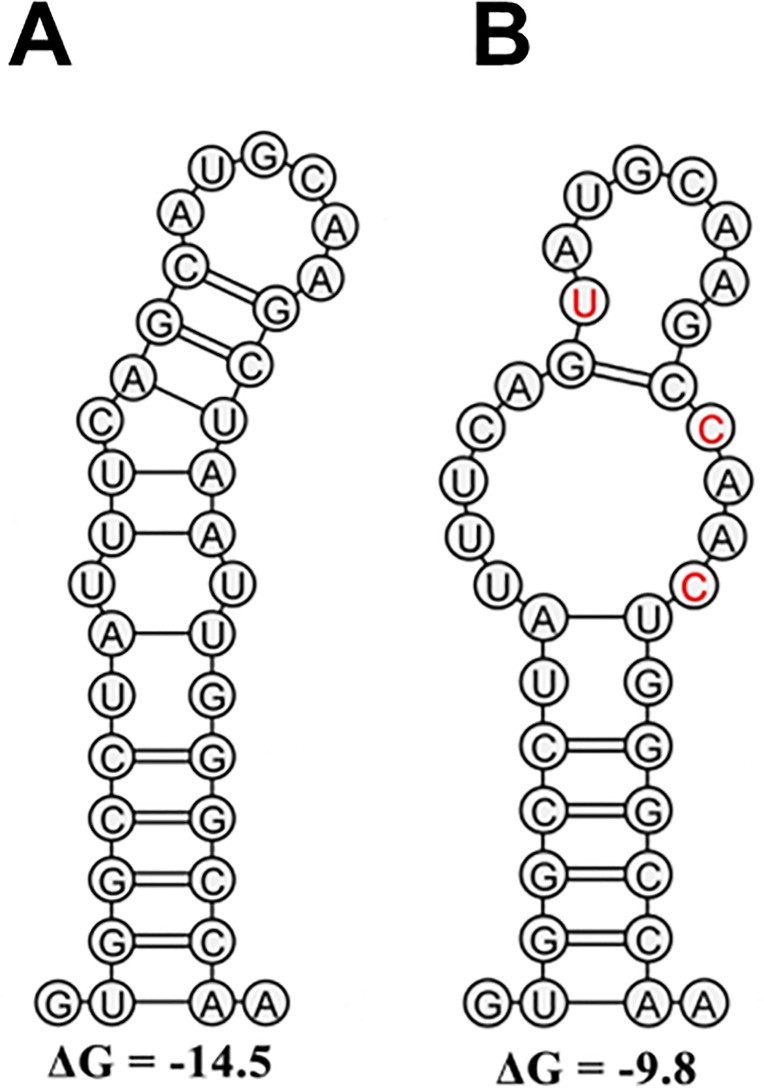
Putative reorganization of conserved structures within the HCV genome over time. The local RNA structure for codons 357–370 were modeled using example sequences from L1a. (A) L1a dominant sequence motif from sample 1. (B) L1a dominant sequence motif from sample 8. Whereas the mutations observed led to an overall decrease in the minimum free energy of the structure, it is the cumulative contribution of mutations across the length of the amplicon that determine overall stability. The predicted ΔG for the full length amplicon was -93.94 and -100.4 for the dominant sequence motifs from sample 1 and 8, respectively. Synonymous mutations at codons 363, 366 and 367 are shown in red.

Discrete RNA structures have been suggested to participate in innate immune evasion and contribute to overall viral fitness [18–20]. Similar secondary structures were predicted within our sequence set. SL1412 as described by Pirakitikulr and colleagues (2016), spans codons 357–371 of the amplicon (Fig 7) [18]. This region is implicated in significant synonymous-synonymous interactions, specifically codons 166 and 167 (Fig 3).

Using the dominant L1a group A and group B sequences as our reference input, the ΔG free energy of the stem loop structure was reduced by the mutations, yet the overall ΔG free energy prediction for the full amplicon was increased. Overall, the impact of individual mutations are unlikely to influence secondary structures which may account for the lack of significant co-variation observed within the L1a group A and B and L1b group B HVR1 sequence subsets. Rather the cumulative effects of mutations across the length of the sequence are the determining factor in genome stability.

## Discussion

The use of a well characterized data set spanning 10 years of treatment naïve, chronic HCV infection facilitated the study and comparison of epistatic mutations within co-circulating virus populations. Of the three virus populations described, L1a and L1b exhibited significant evidence of epistatic evolution. Whereas L1a co-variation was ordered, and both HVR1 subgroups of variants had periods of sample dominance, L1b co-evolution was irregular. The accommodation of an in-frame insertion to the HVR1 of L1b sequences is credited for this difference. L2 sequences had the highest number of invariant sites overall. The L2 sequence subset had the fewest number of unique sequences available for analysis and this in part may account for the higher proportion of invariant sites. It may also reflect an inability to tolerate deleterious mutations, resulting in the efficient removal of low fitness variants from the population [21].

It was apparent at various points of the study, that the insertion event defined the L1b phenotype. The first example was the codon usage bias of L1b sequences. It is recognized that codon usage bias among RNA viruses is largely determined by mutation pressures rather than tRNA availability within the host cell and the viruses own genome nucleotide content [22, 23]. Innate immune recognition of short sequence patterns is also believed to influence codon usage bias in HCV [24]. Consistently, our data shows L1b had lower ENC values than for other (sub-)lineages (Fig 2). The ENC values observed in the current study are considerably lower than those reported by Belalov and Lukashev (2013) who documented a range of 38.2–58.3 for 29 animal RNA viruses [23]. This is likely due to the differing sequence lengths used (full length genomes as opposed to a 294 bp amplicon). Nevertheless, the values for L1b are suggestive of additional constraints placed on L1b sequences.

The ENC results may also have been influenced by the presence of secondary structures in this region of the genome [25–28]. Given that L1b sequences contained an in-frame 3 bp insertion, it is probable that secondary structures in this region would be initially disrupted by the insertion event. For HIV-1 it is well established that gene junctions are enriched in RNA secondary structures and that such structures facilitate recombination-mediated gene swapping [29]. Conserved secondary RNA structures have also been mapped along the entire length of the HCV genome including elements of the E1/E2 gene junction [18–20]. We have previously documented recombinants within our data set and identified the recombination breakpoints at the E1/E2 gene junction [7, 17]. Nascent RNA structures have been known to stimulate transcriptional pausing or transcript release for some time [30]. Synonymous mutations have the potential to discretely alter these structures influencing the outcome of genomic replication events. Identification of a synonymous mutation stably maintained during infection was demonstrated to increase the stability of the RNA structures [19, 31]. Local RNA structures form part of genome-scaled ordered RNA structures (GORS) and such genome organization can be found in many mammalian RNA viruses [28, 32]. GORS form networks of regulatory structures and an understanding of their roles is the focus of ongoing research [18, 19, 28, 33]. While the cumulative impact to the virion is difficult to define, the analysis of localized structures has identified a number of roles including innate immune masking, fitness, replication and infectivity effects [18, 19, 34].

Specifically, mutations that strengthened and mutations that weakened a stem loop region of the HCV genotype 2 Jc1 infectious clone, encompassing codons 357–371 of the amplicon reported here, significantly diminished and improved infectivity, respectively [18, 35]. This region formed part of the synonymous component of the L1a epistatic network (Fig 3). Additionally, the synonymous-synonymous pairing of codons 366 and 367 was the most significant of all synonymous-synonymous recorded for L1a. It is probable that the pairwise change was directly linked to the predicted stem loop structure of this region (Fig 7).

We observed significant nonsynonymous co-variation across the E1/E2 gene junction at discrete sites (most notably codon 349 for both L1a and L1b). Extensive, genome-wide networks of HCV protein co-evolution have been described which suggest intra-cellular interactions [36, 37]. Weak evidence of predicted co-variation between the E1 trans-membrane domain and E2 HVR1 has previously been presented [36]. Together, the data suggests intra-cellular processes between viral proteins will result in the development of vast co-variation networks. Ongoing evolution over extended periods in a single host may allow for the linkage of discrete site to these networks additively, enhancing overall fitness.

Whether the net effect of epistasis observed in this study was antagonistic or synergistic can be evidenced from the relative dominance of variant groups. The deterministic facet of co-variation within the L1a sequence set was nonsynonymous mutations at the HVR1 through required preservation of the regions’ physio-chemical properties [38, 39]. The consequent genetic drift was accompanied by periodic dominance of the newly emergent variants (S1 Fig). Here epistasis was contributing to adaptation and, cumulatively, new variants emerged to dominate. Conversely, the prevalence of synonymous sites participating in co-variation with nonsynonymous change at the HVR1 suggests compensatory evolution at secondary sites within the L1b group A sequence set (Figs 3 and 4). This was compounded by the disproportionate odds of a mutation participating in a co-evolutionary event at one site over the other (Fig 5 and S5 Table). The co-variation profile of L1b demonstrated that group A variants were unfit and such mutations were likely antagonistic. L1b group B variants were fit, yet could not overcome eventual IgG-targeting through HVR1 genetic drift (Fig 6 and S1 Fig).

The perception that synonymous change equates to ‘silent’ change is false [9, 10, 12, 40]. In this report, we add to observations that synonymous substitutions occurring amongst related viral variants may hold answers with respect to viral fitness and evolutionary strategies [9, 14, 41]. Building on previous definitions of virus lineage, dominance removal and emergence, we have demonstrated clear evidence of directed synonymous change across the E1/E2 gene junction of HCV [7, 17]. Increasingly, longitudinal reports of HCV infection over decades have identified collapses in sequence heterogeneity, yet viral infection persists. Our data indicates that the acquisition and accommodation of host adaptations over prolonged periods is, in part, maintained and governed at the level of synonymous mutation.

## Materials and Methods

### Sample data set and preparation

The primary data set on which this study was based has been previously reported [7]. The fasta sequence file used to generate the results in this paper has been deposited on figshare (https://figshare.com/articles/RL1-10_fas/4223799). Those sequences above a sample specific frequency cut off of 0.1% were retained for downstream analysis and known recombinant sequences were removed [7]. Primer sites at the 5′- and 3′-ends of all sequences were clipped resulting in a final fragment of length 294 bp available for analysis, corresponding to positions 1311–1604 of reference genotype 1a strain H77 (Genbank accession: AF009606). The sequence data set was analysed by (sub-)lineage.

### Reference data

A total of 50 unrelated complete genotype 4 genome sequences were retrieved from the Los Alamos National Laboratory HCV sequence database [42]. All sequences were aligned and trimmed to match the 294 bp (297 bp for L1b) E1/E2 region using MEGA6 [43].

### Identification of epistatic linkages

Sequences were grouped based on phylogenetic analysis into four sets, namely L1a, L1b, L1c and L2 [7]. Initially the most frequently occurring codon by site (from nonredundant sequence data across all ten samples) was identified and set as the baseline from which change to an alternate codon could be identified. Within each sequence where codon change occurred, simultaneous site changes with other within-sequence sites were identified and the overall inter-sequence observances of matches enumerated.

The number of simultaneous changes across all sequences is considered to have a binominal distribution where 1 applies to a change occurring at both site A and site B simultaneously, and 0 where there is change at site A but not site B, there is change at site B but not site A or there is no change at site A and site B. The observed probability of a change occurring at each individual site (i.e. P(A), P(B), P(C), P(D)……) was calculated by enumerating the number of observed changes at that site and dividing by the number of potential changes (the number of unique sequences present). In the absence of co-evolutionary constraints, change at individual sites was hypothesised to occur independently of change at subsequent sites. Therefore, the probability of change occurring simultaneously at site A and site B is the product of the individual probabilities of a change occurring at site A and site B. The binomial parameter, *p*, is estimated to be this product of the probability of a change occurring at site A and the probability of change occurring at site B and the binomial parameter *n* is the number of sequences available for analysis.

The observed probability of a change occurring simultaneously at any two sites, *p*_*obs*_, was calculated by enumerating the number of observed simultaneous changes at that pair of sites and dividing by the number of potential changes (the number of unique sequences present). The probability of simultaneous site change occurring independently, *p*, was then compared against the observed probability of simultaneous site change, *p*_*obs*_ using the binomial test. This test was carried out for all site pairs in the sequence. An important caveat to the assumption of independent between-site co-variation is that mutational change at the HVR1 is known to require physio-chemical conservation of the region [38, 39]. This informed post-hoc analysis of the data.

The resultant p-value from the binomial test of mutual change occurring at two sites was adjusted to account for Type I errors generated by multiple comparisons using the False Discovery Rate (FDR) procedure [44]. For the purposes of our analysis, all FDR-adjusted p-values <0.01 were deemed significant. Furthermore, the strength of any co-variation was ordered by the associated FDR-adjusted p-value (S4 and S5 Tables). Analyses were performed using R version 3.1.3 [45].

Downstream analysis of co-varying pairs identified by the above procedure is presented in terms of sites mutating nonsynonymously and synonymously. Nonsynonymous sites are defined as an amino acid change occurring in one or more sequences over all sequences analyzed. Synonymous sites have undergone synonymous change exclusively over all sequences analyzed.

### Bioinformatic analyses

Phylogenetic analysis of the L1b sub-lineage was performed using MEGA6 [43]. First, the sequence data was analysed using jModelTest to determine an appropriate model of nucleotide substitution [46]. A general time-reversible model was chosen with gamma-distributed and invariant sites (GTR+G+I). Bootstrap resampling (1000 datasets) of the multiple alignments was used to test the statistical robustness of the trees.

The effective number of codons (ENC) was enumerated using the “chips” module hosted by EMBOSS [47]. ENC is an intuitive measure of codon usage bias. Values range from a low of 20 (only one codon per amino acid used) to a high of 61 (i.e. the use of alternative synonymous codons is equally likely).

RNA secondary structures and free energies were predicted using mfold [48]. Structures were rendered using VARNA [49].

## Supporting Information

S1 Fig Within-sublineage codon fixation during the study timeframe. L1a and L1b sequence sets were split into two groups based on HVR1 amino acid motif. (A) Left panel: For the purposes of this analysis, a site is designated as fixed (black) when a single codon accounts for all the sequences in that sample and all subsequent samples thereafter. Samples with absent or insufficient sequence data are shaded grey. The E1/E2 gene junction and the last codon of the HVR1 are identified by a solid red line and a dashed red line, respectively. Samples with absent or insufficient sequence data (less than two unique sequences) are shaded as grey horizontal bars. Tick marks along the X-axis identify each codon position of the amplicon sequence. Right panel: The sample specific frequency of each (sub-)lineage. (B) Consensus HVR1 motif sequences for L1a, groups A and B, and L1b, groups A and B.
(TIF)

S1 Table Initial sequence numbers and lineage frequencies (XLSX)

S2 Table Frequency of codon changes among co-evolutionary sites observed in L1a sequences (XLSX)

S3 Table Frequency of codon changes among co-evolutionary sites observed in L1b sequences (XLSX)

S4 Table Odds of participation in a significant co-evolutionary events in L1a sequences ranked in order of p-value (XLSX)

S5 Table Odds of participation in a significant co-evolutionary events in L1b sequences ranked in order of p-value (XLSX)
