# Supplementary figures and images for: Synonymous Co-Variation across the E1/E2 Gene Junction of Hepatitis C Virus Defines Virion Fitness
Source: PLoS One. 2016 Nov 23;11(11):e0167089. doi: 10.1371/journal.pone.0167089 (PMC5120871; doi:10.1371/journal.pone.0167089)

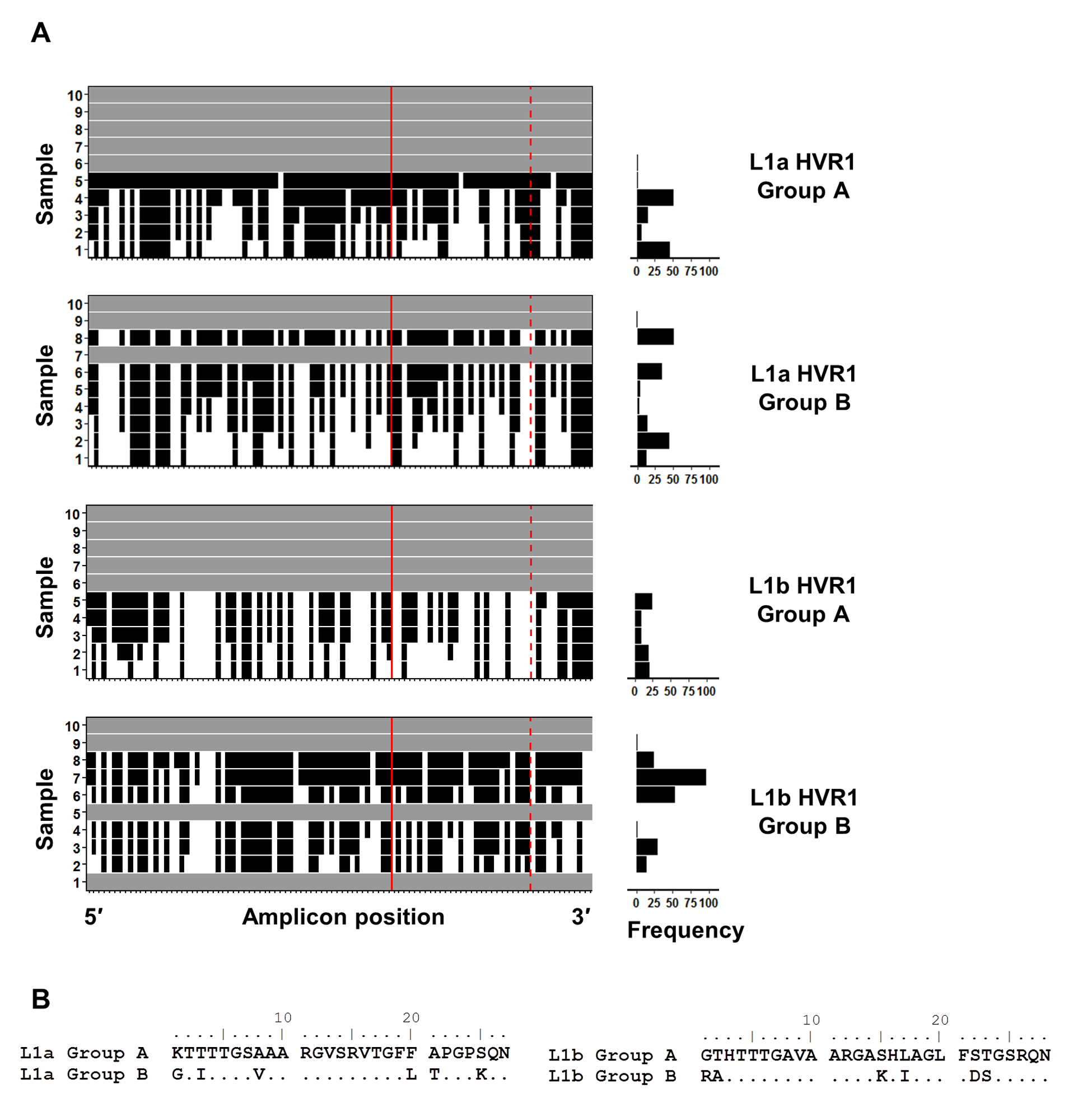

Supplement: S1 Fig — L1a and L1b sequence sets were split into two groups based on HVR1 amino acid motif. (A) Left panel: For the purposes of this analysis, a site is designated as fixed (black) when a single codon accounts for all the sequences in that sample and all subsequent samples thereafter. Samples with absent or insufficient sequence data are shaded grey. The E1/E2 gene junction and the last codon of the HVR1 are identified by a solid red line and a dashed red line, respectively. Samples with absent or insufficient sequence data (less than two unique sequences) are shaded as grey horizontal bars. Tick marks along the X-axis identify each codon position of the amplicon sequence. Right panel: The sample specific frequency of each (sub-)lineage. (B) Consensus HVR1 motif sequences for L1a, groups A and B, and L1b, groups A and B. (TIF) [file pone.0167089.s001.tif]
